# On the screening, diagnosis, and treatment of adolescent and adult ADHD patients with comorbid substance use: international guidelines

**DOI:** 10.1192/j.eurpsy.2023.52

**Published:** 2023-07-19

**Authors:** C. L. Crunelle

**Affiliations:** Psychiatry, Vrije Universiteit Brussel, Brussels, Belgium

## Abstract

**Abstract:**

Attention deficit/hyperactivity disorder (ADHD) often co-occurs with substance use disorders (SUD). Together, these disorders are associated with significantly more burden for patients and society, than each alone. Patients with SUD and underlying ADHD have more complex SUD and have more poly-substance use compared with SUD patients without ADHD. A correct identification of ADHD in adult and adolescent individuals with SUD remains important regarding treatment, treatment effectiveness, and treatment retention.

Several screening tools are available and have been validated in individuals with ADHD and comorbid SUD. It is highly recommended that these are used routinely, followed by an ADHD diagnostic process initiated as soon as possible. While several treatment options are accessible, randomized controlled trials show only limited effect sizes of standard pharmacotherapy in adult and adolescent ADHD patients with comorbid SUD. Simultaneous and integrated treatment, with a combination of pharmacotherapy and psychotherapy and for both ADHD and SUD, should preferably be initiated.

We present an overview of the current international guidelines on screening, diagnosis and treatment of ADHD adults and adolescents with comorbid substance use disorders.

**Disclosure of Interest:**

None Declared

